# Complete Genome Sequence of Reticuloendotheliosis Virus Strain MD-2, Isolated from a Contaminated Turkey Herpesvirus Vaccine

**DOI:** 10.1128/genomeA.00785-13

**Published:** 2013-10-03

**Authors:** Junping Li, Chenghuai Yang, Qihong Li, Huijiao Li, Yecai Xia, Dan Liu, Kangzhen Yu, Hanchun Yang

**Affiliations:** Key Laboratory of Animal Epidemiology and Zoonosis of Ministry of Agriculture, College of Veterinary Medicine and State Key Laboratory of Agrobiotechnology, China Agricultural University, Beijing, People’s Republic of Chinaa; China Institute of Veterinary Drug Control, Beijing, People’s Republic of Chinab; Ministry of Agriculture, Beijing, People’s Republic of Chinac

## Abstract

Here, we present the complete genomic sequence of a reticuloendotheliosis virus (REV) isolated from a contaminated turkey herpesvirus (HVT) vaccine. This report will be helpful for epidemiological studies on REV infection in avian flocks.

## GENOME ANNOUNCEMENT

Reticuloendotheliosis virus (REV), classified as a gammaretrovirus, causes immunosuppression, running disease, and lymphomas. The representative strains of REV include the defective REV-T and the nondefective REV-A, the spleen necrosis virus (SNV), duck infectious anemia virus, and chick syncytial virus (CSV) (1). All REV isolates are antigenically related to each other and have a wide avian host range that includes chickens, turkeys, ducks, geese, pheasants, peafowl, Japanese quail, and prairie chickens (2–4). REV has been found as a contaminant in commercial vaccines in some countries as early as the 1970s (5, 6). In recent years, there were cases of vaccines contaminated with REV in China and other countries (7–9). However, there is no report about the complete genome sequence of REV isolated from a contaminated vaccine.

REV strain MD-2 was isolated from a batch of commercial freeze-dried turkey herpesvirus vaccines in 2007 and propagated in chicken embryo fibroblasts (CEF). According to the Wizard Genomic DNA purification kit (Promega, Madison, WI) protocol, total genomic DNA was extracted from infected CEF and used as template for proviral DNA amplification. Seven pairs of primers were designed in overlapping regions for PCR amplification. The 7 amplified PCR products were purified and cloned into pMD18-T vector (Takara, Dalian, China) and were sequenced by BGI (Beijing, China). The genomic sequence was assembled using the SeqMan function in the DNAStar sequence analysis software (DNAStar, Inc., Madison, WI). The long terminal repeat (LTR) sequence was deduced based on the notion that both LTRs are identical in the REV proviral genome.

The proviral genome cDNA is 8,284 nucleotides long and exhibits a genetic organization characteristic of replication-competent gammaretroviruses. The proteins were deduced according to the features of gammaretroviruses and previous studies (10, 11). The *pol* gene is situated in the continuous open reading frame (ORF) as *gag* and translated gag-pol polyprotein via termination suppression of an amber stop codon (12). The *env* gene is located in an independent ORF, and expression of the *env* gene is driven by a spliced mRNA. The gag precursor protein is 499 amino acids long and is cleaved into 4 structural proteins, matrix (MA) extending from amino acids 2 to 114, p18 from amino acids 115 to 199, capsid (CA) from amino acids 200 to 442, and nucleocapsid domain (NC) from amino acids 443 to 494. The *env* precursor is 587 amino acids long with a signal peptide of 36 amino acids located in the NH2 terminal region, and it is cleaved by cellular furin on amino acid 398 to produce the mature surface (SU) and transmembrane (TM) proteins. The genome of MD-2 strain is most similar to that of strain HLJR0901, isolated in 2009 in China (13), with 99.9% identity. This report will be helpful for epidemiological study investigation on REV infection in an avian flocks.

### Nucleotide sequence accession number.

The complete genome sequence of the REV strain MD-2 is available in GenBank under the accession no. JX912710.
